# Concentrations of HMGB1 and Hsp70 of healthy subjects in upper and lower airway: Literature Review and Meta-analysis

**DOI:** 10.7150/ijms.53500

**Published:** 2021-02-18

**Authors:** Hyun Jin Min, Kyung Soo Kim, Geun Joo Choi, Hyun Kang, Fletcher A. White

**Affiliations:** 1 Department of Otorhinolaryngology-Head and Neck Surgery, Chung-Ang University College of Medicine, Seoul, Korea.; 2 Department of Anesthesiology and Pain Medicine, Chung-Ang University College of Medicine, Seoul, Korea.; 3 Department of Anesthesia, Indiana University School of Medicine, IN, USA.

**Keywords:** high-mobility group box 1 protein, heat-shock protein 70, reference ranges, biomarkers, meta-analysis

## Abstract

Although high-mobility group box 1 and heat-shock protein 70 are implicated in airway diseases and suggested as relevant diagnostic biomarkers, their control concentrations in the airways have not yet been determined. This study aimed to evaluate concentration of healthy subjects for both these proteins in the upper and lower airways via meta-analysis.

We searched MEDLINE, EMBASE, and Google Scholar for articles describing concentration of healthy subjects for these proteins. Data from healthy populations were combined using a random-effects model, and subgroup and sensitivity analyses were performed to determine between-study heterogeneity. We analyzed 22 studies involving 485 patients.

Concentration of healthy subjects of high-mobility group box 1 and heat-shock protein 70 varied from “not detected” to 326.13 ng/mL and from 0.20 pg/mL to 9240.00 pg/mL, respectively, with the values showing significant heterogeneity. Subgroup analysis for high-mobility group box 1 revealed 13.63 ng/mL (95% CI 12.13-15.14), 100.31 ng/mL (95% CI -31.28-231.91), 9.54 ng/mL (95% CI 8.91-10.17), and 65.82 ng/mL (95% CI 55.51-76.14) for the lower airway, upper airway, pediatric populations, and adults, respectively, whereas that for heat-shock protein 70 revealed 20.58 pg/mL (95% CI 7.87-33.29) for the lower airway and 9240.00 ±11820 pg/mL for the upper airway. Although concentrations of healthy subjects of these proteins varied in the upper and lower airways, the levels of both these proteins were higher in the upper airway than in the lower airway, and these concentrations differed according to the age and sampling procedure.

Our findings support the further evaluation of these proteins as biomarkers for airway-related diseases.

## Introduction

The airway mucosa is important as it serves as the first line of host defense in the innate immune system 1,2. Several structurally unrelated damage-associated molecular patterns (DAMPs) have been identified since the introduction of “the danger model” 1, with these molecules being released from host cells in response to cellular stress or tissue injury. DAMPS are capable of inducing inflammatory responses by activating the innate immune system as important mediators of the innate immune response 2.

High-mobility group box 1 (HMGB1) is an actively studied DAMP molecule. HMGB1 functions as a cytokine following its extracellular release from injured cells, activated sensory neurons 3, or cells associated with the immune system (macrophages and dendritic cells) 4. It is a nuclear protein that regulates gene expression by binding to DNA in a non-sequence-specific manner, with its affinity for DNA being regulated by phosphorylation and acetylation 4. In the extracellular compartment, HMGB1 binds to receptors, including toll-like receptor (TLR) 2, TLR4, and receptor for advanced glycation end products (RAGE) 5,6, which triggers the activation of proinflammatory signaling pathways and exerts pleiotropic effects depending on the cell type 5.

Recently, heat-shock protein (Hsp) 70 was suggested as another DAMP molecule. Unlike HMGB1, Hsp70 is present in the cytoplasm and functions as a highly conserved molecular chaperone by facilitating intracellular protein folding and the degradation of misfolded proteins. Upon its extracellular release during cell stress or injury, Hsp70 activates both TLR2 and TLR4 signaling 2,7.

We previously reported the expression of both HMGB1 and Hsp70 in upper airway epithelial cells and their release into the extracellular area 5,8-10. Additionally, previous studies have reported HMGB1 and Hsp70 expression and their roles as DAMPs in airways 11-13. Although multiple studies have demonstrated that HMGB1 and Hsp70 function as DAMPs and suggest their importance as diagnostic markers in upper and lower airway diseases, their concentration of healthy subjects under physiological conditions remain unknown. Although the upper and lower airways are generally considered to be united, their immunological conditions are suggested to be different 14. This suggests that the concentrations of HMGB1 and Hsp70 and their potential immunological roles in the upper and lower airways may also differ. In this study, we performed a meta-analysis to determine the concentration of healthy subjects of HMGB1 and Hsp70 in the upper and lower airways and analyzed their differences through a literature review.

## Methods

We developed and prospectively registered the study protocol using PROSPERO (registration number: CRD42018105478; www.crd.york.ac.uk/PROSPERO). This study was performed according to the Meta-analysis of Observational Studies in Epidemiology guidelines 15 and reported according to the Preferred Reporting Items for Systematic reviews and Meta-Analysis guidelines 16.

### Inclusion and Exclusion Criteria

All randomized controlled studies, cohort studies, case-control studies, including nested case-control studies, and cross-sectional studies reporting normal values of HMGB1 and Hsp70 in the upper and lower airways of healthy populations were included. We included only studies that performed enzyme-linked immunosorbent assay (ELISA) to objectively quantify the HMGB1 and Hsp70 levels in nasal lavage (NAL) or bronchoalveolar (BAL) fluids or induced sputum.

The exclusion criteria were: 1) review articles, case reports, case series, letters to the editor, commentaries, proceedings, abstracts without complete published articles, laboratory studies, and any other non-relevant studies; 2) studies reporting HMGB1 and Hsp70 levels in patients with a disease; 3) studies reporting HMGB1 and Hsp70 levels in animals; 4) studies reporting HMGB1 and Hsp70 levels in regions other than the airways; and 5) studies performing a relative comparison of HMGB1 and Hsp70 concentrations using western blot, real-time polymerase chain reaction, or immunohistochemistry. No language limitations or date restrictions were imposed.

### Information Sources and Search Strategy

Two authors (HJM and HK) independently performed a database search using OVID-MEDLINE, EMBASE, and Google Scholar in August 2018 and updated the search results in March 2019. Additionally, the reference lists of the identified studies and eligible articles were searched manually. The search strategy, which included a combination of free text, medical subject headings, and EMBASE subject headings, is described in Appendix S1 in the Supplementary Information.

### Study Selection

Two authors (HJM and KSK) independently scanned the titles and abstracts of the reports identified via the search strategies and retrieved the full paper upon determining the eligibility of the study. Potentially relevant studies selected by at least one author were retrieved, and the full-text versions were evaluated. Two authors (HJM and KSK) discussed the relevance of each study to arrive at a consensus regarding study inclusion or exclusion. Disagreements were settled via discussion with a third investigator (HK). None of the authors had any objection to the final results.

### Data Extraction

All interrelated data from the included studies were independently extracted, entered in a standardized form by two authors (HJM and KSK), and crosschecked. When an agreement could not be reached, the dispute was resolved with the aid of a third investigator (HK). The standardized form included the title, name of the first author, name of the journal, year of publication, study design, the country where the study was performed, sex, age, number of patients, history of smoking, the location where the sample was drawn, sampling method, analysis method, normal range of Hsp70, and normal range of HMGB1. The data were initially extracted from the tables or text. In cases of missing or incomplete data, an attempt was made to contact the authors to obtain the relevant information. Whenever the reported data were in the form of a median (P_25_-P_75_), median (range), or mean (standard error of the mean), the mean and standard deviation were calculated from these values 17,18. For data presented as figures rather than numbers 19-23, the open-source software Plot Digitizer (v.2.6.8; http://plotdigitizer.sourceforge.net) was used to extract the numbers.

### Statistical Analysis

Data were combined using a random-effects model, and subgroup and sensitivity analyses were performed to determine between-study heterogeneity. We performed all analyses using Stata SE (v.15.0; StataCorp, College Station, TX, USA) and comprehensive meta-analysis software (v.2.0; Biostat, Englewood, NJ, USA). The methods used for statistical analysis are described in detail in Appendix S1 in the Supplementary Information.

## Results

### Search Results

**Figure 1:**
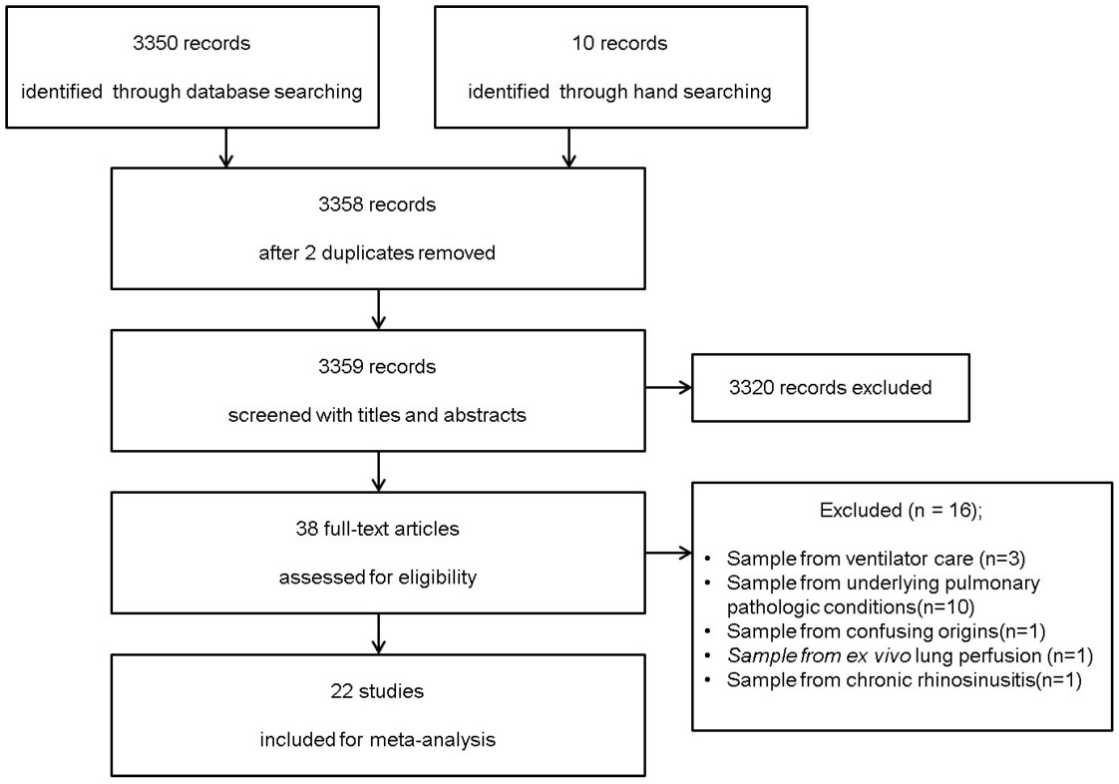
Flow diagram of the search process and the inclusion and exclusion criteria for the meta-analysis.

We identified 3,350 potentially relevant studies from the database search (OVID-MEDLINE, EMBASE, and Google Scholar) and 10 studies from a manual search. After excluding 2 duplicates, 3,358 records were screened based on their titles and abstracts. Of these, 3320 studies were excluded because they did not belong in the area of interest. The full texts of the remaining 38 studies were reviewed in detail. Sixteen studies were excluded for the following reasons: 1) control subjects were on ventilator care (*n* = 3) 24-26; 2) BAL fluid was obtained from subjects with underlying pulmonary pathologic conditions (*n* = 10) 27-36; 3) there was confusion regarding whether the control subjects were healthy or had non-cystic fibrosis with recurrent infections (*n* = 1); 4) BAL fluid was harvested during *ex vivo* lung perfusion (*n* = 1); 5) NAL fluid was obtained from patients with chronic rhinosinusitis (*n* = 1) 9. Therefore, 22 studies comprising 485 subjects met the inclusion criteria and were included in this systematic review and meta-analysis (Figure 1). The kappa value for selecting articles between the two reviewers was 0.756.

### Description of the Studies

Table 1 contains a description and summary of each study. Five studies reported HMGB1 and Hsp70 in the upper airway 5,8,19,22,37, and 17 studies reported them in the lower airway 20,21,23,25,30,34,38-48. In 5 studies, NAL fluid was obtained from the upper airway 5,8,19,22,37, whereas 16 studies used induced sputum or BAL fluid from the lower airway 20,21,23,25,34,38-48, with 1 study describing the collected BAL fluid as epithelial-lining fluid 30. Ten studies were conducted in Eastern Asia 5,8,22,30,34,38,39,41,45,48, eight in Europe 19,21,25,37,40,42,43,46, one in the United Kingdom 20, one in Australia 44, and the remaining two in the United States 23,47.

### Analysis of HMGB1 concentrations according to location

**Figure 2:**
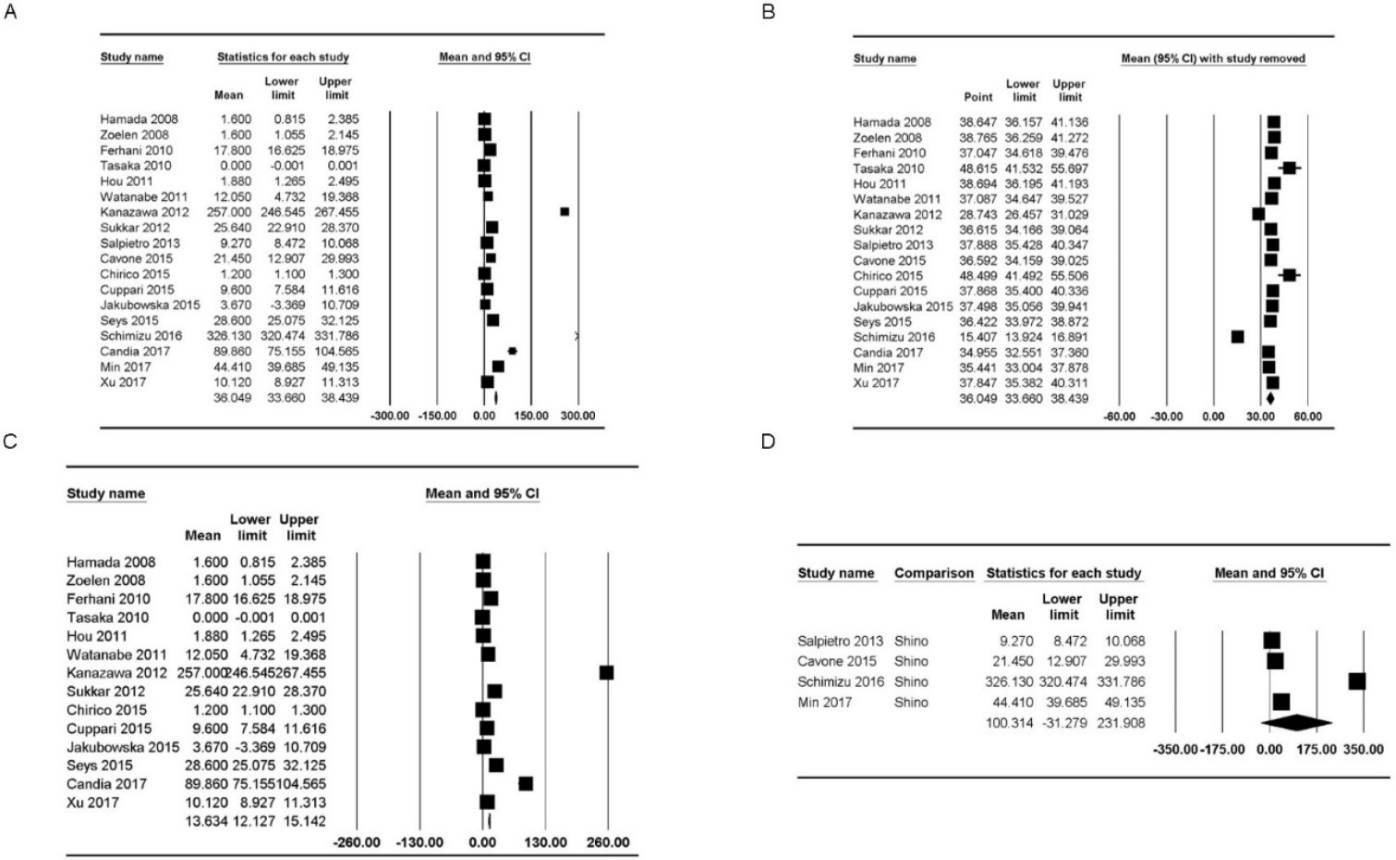
** Forest plots of the concentration of healthy subjects of HMGB1 in the upper and lower airways.** (A) Plot for HMGB1 in the upper and lower airways. (B) Plot for sensitivity analysis, excluding one study at a time, for HMGB1 in the upper and lower airways. (C, D) Plots for HMGB1 in the (C) lower and (D) upper airways. The figure depicts individual trials as filled squares with sizes relative to the sample size and the 95% confidence interval of the difference as the solid line. The diamond shape indicates the pooled estimate and uncertainty for the combined effect.

HMGB1 concentrations were reported in 18 studies 5,19-22,25,30,34,37-46. The concentration of healthy subjects in the upper and lower airways varied from “not detected” to 326.13 ng/mL [mean: 36.05, 95% confidence interval (CI): 33.66-38.44] but showed significant heterogeneity (P_chi_^2^ < 0.001; I^2^ = 99.91) (Figure 2A). Additionally, we performed sensitivity analysis by excluding one study at a time (Figure 2B). After excluding studies by Tasaka et al. 34 and Chirico et al. 43, the HMGB1 concentration range increased substantially (mean: 48.62 ng/mL, 95% CI: 41.53-55.69; mean: 48.50 ng/mL, 95% CI: 41.49-55.51), and after excluding studies by Kanazawa et al. 30 and Shimizu et al. 22, the HMGB1 concentration range decreased substantially (mean: 28.74 ng/mL, 95% CI: 24.46-31.03; mean: 15.41 ng/mL, 95% CI: 13.92-16.89); however, the heterogeneity remained significant (P_chi_^2^ < 0.001, I^2^ = 99.91; P_chi_^2^ < 0.001, I^2^ = 99.91; P_chi_^2^ < 0.001, I^2^ = 99.90; and P_chi_^2^ < 0.001, I^2^ = 99.73, respectively).

According to meta-regression, the location where the sample was drawn [coefficient (Coef) = 67.49, 95% CI: -40.68-175.66; p = .205], the ELISA method (Coef = 38.43, 95% CI: -59.97-136.83; p = .420), and the age category (Coef = 57.76, 95% CI: -81.48-197.02; p = .420) were unlikely to be sources of heterogeneity (Table 2).

### HMGB1 concentrations in the lower airway

HMGB1 concentrations in the lower airway were reported in 14 studies 20,21,25,30,34,38-46. The concentration of healthy subjects of HMGB1 varied from “not detected” to 257.00 ng/mL (mean: 13.63 ng/mL, 95% CI: 12.13-15.14) but showed significant heterogeneity (P_chi_^2^ < 0.001, I^2^ = 99.74) (Figure 2C). Sensitivity analysis after excluding studies by Tasaka et al. 34 and Chirico et al. 43 showed that the HMGB1 concentration range increased substantially (mean: 27.87 ng/mL, 95% CI: 23.52-32.22; mean: 27.96 ng/mL, 95% CI: 23.53-32.38), and sensitivity analysis after excluding the study by Kanazawa et al. 30 showed that the HMGB1 concentration range decreased substantially (mean: 8.45 ng/mL, 95% CI: 7.29-9.60); however, the heterogeneity remained significant (P_chi_^2^ < 0.001, I^2^ = 99.70; P_chi_^2^ < 0.001, I^2^ = 99.73; and P_chi_^2^ < 0.001, I^2^ = 99.54, respectively).

According to subgroup analysis based on the sampling method, concentration of healthy subjects of HMGB1 were 6.19 ng/mL (95% CI: 1.84-10.54; P_chi_^2^ < 0.001; I^2^ = 99.67) when BAL was used 20,21,23,25,34,38,40,41,45-47 and 17.62 ng/mL (95% CI: 13.04-22.20; P_chi_^2^ < 0.001; I^2^ = 99.21) when induced sputum was used 39,42-44,48.

### HMGB1 concentrations in the upper airway

The HMGB1 concentrations in the upper airway of healthy populations were reported in four studies 5,19,22,37 and ranged from 9.27 ng/mL to 326.13 ng/mL (mean: 100.31 ng/mL, 95% CI: -31.28-231.91) but showed significant heterogeneity (P_chi_^2^ < 0.001, I^2^ = 99.98) (Figure 2D). Sensitivity analysis after excluding the study by Shimizu et al. 22 showed that the HMGB1 concentration range decreased, though the heterogeneity remained significant (mean: 25.01 ng/mL, 95% CI: 10.37-55.37; P_chi_^2^ < 0.001; I^2^ = 99.06).

### Analysis of Hsp70 concentrations according to location

**Figure 3:**
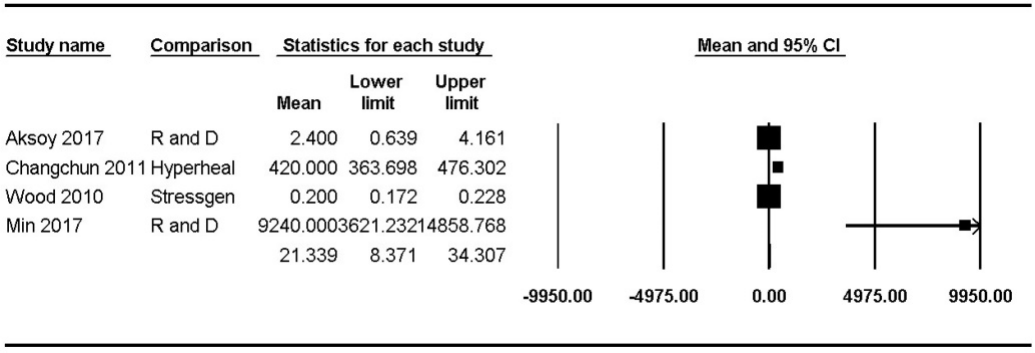
** Forest plots of the concentration of healthy subjects of Hsp70 in the upper and lower airways.** The figure depicts individual trials as filled squares with sizes relative to the sample size and the 95% confidence interval of the difference as the solid line. The diamond shape indicates the pooled estimate and uncertainty for the combined effect.

Hsp70 concentrations were reported in four studies 8,23,47,48 and ranged from 0.20 pg/mL to 9240.00 pg/mL (mean: 21.34, 95% CI: 8.37-34.31) but showed significant heterogeneity (P_chi_^2^ < 0.001, I^2^ = 98.70) (Figure 3).

### Hsp70 concentrations in the upper and lower airways

Hsp70 concentrations in the lower airway of healthy populations were reported in three studies 23,47,48 and ranged from 0.2 pg/mL to 420.00 pg/mL (mean: 20.58, 95% CI: 7.87-33.29) but showed significant heterogeneity (P_chi_^2^ < 0.001, I^2^ = 99.09). Sensitivity analysis after excluding the study by Hou et al. 48 revealed a substantial decrease in the Hsp70 concentration range, though the heterogeneity remained significant (mean: 1.12, 95% CI: -1.01-3.24; P_chi_^2^ < 0.001; I^2^ = 83.32).

The Hsp70 concentration in the upper airway of healthy populations was reported in only one study (9240 ± 11,820 pg/mL) 8.

## Discussion

DAMPs, such as HMGB1 and Hsp70, are conducive to inflammatory damage associated with inflammatory diseases of the airways 2,49. Because the concentrations of HMGB1 and Hsp70 can be elevated and have been suggested as potential biomarkers in various diseases, verifying the normal control concentrations of these molecules in the airways is essential to assess their efficacy as biomarkers.

This study revealed that HMGB1 and Hsp70 are present in substantial concentrations in the extracellular areas in the upper and lower airways of healthy controls. Analysis of the primary outcome showed that the mean concentration of HMGB1 was 13.63 ng/mL in the lower airway 20,21,25,30,34,36,39-46 and 100.31 ng/mL in the upper airway 5,19,22,37 and that the mean concentration of Hsp70 was 20.58 pg/mL in the lower airway 22,47,48 and 9240.00 pg/mL in the upper airway 8. However, a limited number of studies evaluating the status of these molecules under normal circumstances, especially their status in the upper airway, have been published, making it difficult to accurately determine their concentration of healthy subjects.

The mean concentrations of HMGB1 and Hsp70 were higher in the upper airway than in the lower airway. The accumulated data suggest that inflammatory diseases of the upper and lower airways share a common background, which is sometimes described as “one airway, one disease” 14,50. However, immunological responses in the upper and lower airways are not always identical. Compared with the upper airway mucosa, which is exposed to various microorganisms and toxic materials, the lower airway mucosa is less frequently associated with pathologic microbes 14. Additionally, the epithelial-barrier cells in the upper airway epithelium differ from those in the lower airway 14. Therefore, we hypothesized that the concentrations of DAMPs might differ between the upper and lower airways. In this analysis, the mean concentrations of both HMGB1 and Hsp70 were higher in the upper airway than in the lower airway, although the difference was not statistically significant, which could be attributed to the limited number of studies evaluating these levels. Nevertheless, our findings support the existence of different immunological responses between the upper and lower airways.

This meta-analysis demonstrated different mean concentrations of HMGB1 in induced sputum (17.62 ng/mL) 39,42-44,48 and BAL fluid (6.19 ng/mL) 20,21,23,25,34,38,40,41,45-47. Sputum analysis represents a non-invasive and cost-effective method potentially useful in clinical practice as a disease-screening procedure 49. However, because the mean concentration of HMGB1 was higher in sputum than in BAL fluid, a known control level for HMGB1 is necessary before its consideration as a diagnostic biomarker. Additionally, NAL fluid is more safely and easily collected from patients (i.e., via nasal irrigation) relative to procedures involving tissue harvesting or blood sampling. A review described previous studies evaluating the importance of HMGB1 in upper airway inflammatory diseases, such as rhinosinusitis and allergic rhinitis; however, the analyses were performed using nasal tissue 51. In this analysis, we found only a limited number of studies using NAL fluid 5,8,19,22. Therefore, clinical studies using NAL fluid and a larger cohort should be performed to analyze the potential significance of HMGB1 and Hsp70 concentrations as non-invasive and efficacious biomarkers for diagnosing upper airway diseases. Furthermore, standardized sampling method for airway fluid collection should be set to get control reference range for these potential biomarkers in regional area.

The HMGB1 concentration differed between pediatric patients and adults. The HMGB1 concentration in the upper airway was 9.27 ng/mL (95% CI: -8.47-10.07) in the pediatric population 37 and 160.20 ng/mL (95% CI: -156.58-163.83) in adults 5,22, whereas the HMGB1 concentration in the lower airway was 9.99 ng/mL (95% CI: -8.96-11.01) in pediatric patients 42,45 and 36.35 ng/mL (95% CI: -30.75-41.96) in adults 20,21,25,30,34,38-41,43,44,46. A previous study identified *HMGB1* as a candidate gene involved in aging and age-related diseases in humans 52, with another study reporting changes in its expression during aging in the mouse brain 53. This suggests that the HMGB1 concentration in the upper and lower airways could depend on patient age. Given that NAL fluid and sputum collection represents a safe and simple procedure in pediatric subjects, further evaluation in children is required to compare changes in HMGB1 concentrations according to age.

Additionally, the concentration of healthy subjects of extracellular HMGB1 (ng/mL) were higher than those of Hsp70 (pg/mL) in both airways. Both HMGB1 and Hsp70 are ubiquitous proteins; therefore, the determination of their DAMP-specific functions as inflammatory modulators is potentially important. Interestingly, a study by Tasaka et al. 34 was the only one to report failure to detect HMGB1 in BAL fluid from healthy controls, though they detected HMGB1 in other groups of patients, such as those with autoimmune disease, malignancy, and acquired immunodeficiency syndrome. Moreover, sputum analysis by another study revealed HMGB1 concentrations ranging from 0 ng/mL to 45.2 ng/mL 38. This large concentration range could be the result of the excessive dilation of target samples or instability of the sampling method; therefore, standardized sampling methods are required.

There are limitations to this meta-analysis. First, compared with studies involving pathological conditions, studies evaluating the control concentration of HMGB1 and Hsp70 in regional airways are very limited, especially, in case of Hsp70 in upper airway. Therefore, the relatively small sample size of the included studies might have influenced the heterogeneity of the results. As interaction between HMGB1 and Hsp70 has been reported in previous study 54, large population study considering HMGB1 and Hsp70 at the same time might reveal true control concentrations. Second, in some studies, the authors did not provide original data regarding the actual concentrations. Although we attempted to contact the authors to obtain this original data, we were unsuccessful in some instances. In those cases, we extracted the data regarding the HMGB1 and Hsp70 concentrations using Plot Digitizer. Thrid, we did not separate Hsp70 into subtypes owing to the small sample size of the enrolled studies 47; however, we note that Hsp70 is among the most commonly studied members of the Hsp family and can be sub-grouped accordingly 55. Furthermore, the levels of other Hsps might be worth evaluating in the context of this analysis. Finally, we did not evaluate the effect of smoking history as only a few studies supplied this information 20,30,34,38-40,42,43,48. Taylor et al. 56 suggested that smoke exposure could influence the concentration of HMGB1. Also, specific information of enrolled population such as racial differences or obesity was not considered in this study. Therefore, the effect of specific characteristics such as smoking history, racial difference, and obesity on healthy subjects' of HMGB1 and/or Hsp70 concentration in regional airways should be evaluated further.

## Conclusion

Overall, in this study, although the concentration of healthy subjects of these proteins varied in the upper and lower airways, the levels of both were higher in the upper airway than in lower airway, and the concentration of healthy subjects differed according to the age and sampling procedure. These findings support the further evaluation of these proteins as biomarkers for airway-related diseases.

## Supplementary Material

Supplementary materials.

## Figures and Tables

**Table 1:** Characteristics of the studies

Authors_year	Study design	Sample (upper/lower airway)	Sex (M/F)	Age (years) (SD)^†^	Samples	Outcomes	Test material	Smoking history (pack/year)
Hyun Jin Min_2017	Prospective	NAL (upper airway)	11/7	44.41 ± 19.2	17	HMGB1	Commercial ELISA (IBL by Shino-Test)	ND
Shino Shimizu_2016	Prospective	NAL (upper airway)	2/4	46.16	6	HMGB1	Commercial ELISA (IBL by Shino-Test)	ND
Leonardo Cavone_2015	Prospective	NAL (upper airway)	ND	ND	12	HMGB1	Commercial ELISA (IBL by Shino-Test)	ND
C.Salpietro_2013	Prospective	NAL (upper airway)	42/55	9.8 ± 4.1	97	HMGB1	Commercial ELISA (IBL by Shino-Test)	ND
Leonarda Di Candia_2017	Prospective	Sputum (lower airway)	7/11	53.0 ± 3.2	18	HMGB1	Commercial ELISA (Oxford Biosystems)	0
Tetsuya Watanabe_2011	Prospective	Sputum (lower airway)	7/8	42 (31-54)	15	HMGB1	Commercial ELISA (IBL by Shino-Test)	0
Sadatomo Tasaka_2010	Prospective	BAL (lower airway)	ND	ND	6	HMGB1	Commercial ELISA (IBL by Shino-Test)	0
Changchun Hou_2011	Prospective	Sputum (lower airway)	22/12	44 (25-70)	34	HMGB1	Commercial ELISA (Hyperheal)	20 (12.5-35)
Nassima Ferhani_20101	Prospective	BAL (lower airway)	11/9	55.3 ± 13	20	HMGB1	Commercial ELISA (IBL by Shino-Test)	0
van Zoelen MA_2008	Prospective	BAL (lower airway)	10/0	32.0 ± 2.5	10	HMGB1	ELISA using monoclonal Ab	ND
Naoki Hamada_2008	Prospective	BAL (lower airway)	ND	ND	18	HMGB1	ELISA using monoclonal Ab	ND
S.F. Seys_2015	Prospective	Sputum (lower airway)	7/8	19.2 ± 3.6	15	HMGB1	Commercial ELISA (Mybiosource)	ND
Caterina Cuppari_2015	Prospective	Sputum (lower airway)	20/20	11.0 ± 2.2	40	HMGB1	Commercial ELISA (IBL by Shino-Test)	ND
V. Chirico_2015	Prospective	Sputum (lower airway)	15/15	23.2 ± 11.3	30	HMGB1	Commercial ELISA (IBL by Shino-Test)	0
Hiroshi Kanazawa_2012	Prospective	ELF (lower airway)	11/3	66 (64-70)	14	HMGB1	Commercial ELISA (IBL by Shino-Test)	0
M.B. Sukkar_2012	Prospective	Sputum (lower airway)	ND	53 ± 21	18	HMGB1	Commercial ELISA (IBL by Shino-Test)	ND
Hui Xu_2017	Prospective	BAL (lower airway)	12/18	3.6 ± 1.1	30	HMGB1	Commercial ELISA (Chuangxiang Biotech)	ND
K. Jakubowska_2015	Prospective	BAL (lower airway)	13/2	60.1 ± 5.0	15	HMGB1	Commercial ELISA (IBL by Shino-Test)	ND
Mark O. Aksoy_2017	Prospective	BAL (lower airway)	8/3	51 ± 3	11	HspA5	Commercial ELISA (R&D Systems)	0
Changchun Hou_2011	Prospective	Sputum (lower airway)	22/12	39 (24-70)	34	Hsp70	Commercial ELISA (Hyperheal)	0 (0-30)
Karen L. Wood_2010	Prospective	BAL (lower airway)	4/4	ND	8	Hsp27, Hsp60, Hsp70	Commercial ELISA (Stressgen)	ND
Hyun Jin Min_2017	Prospective	NAL (upper airway)	12/5	28.9 ± 9.2	17	Hsp70	Commercial ELISA (R&D Systems)	ND

^†^Data are presented as the means ± standard deviations or medians (Q1-Q3). ND, not described.

**Table 2:** Meta-regression for high-mobility group box 1

Category	Coef	95% CI	*p*
Lower versus upper	67.49	-40.68-175.66	0.205
Shino versus without Shino	38.43	-59.97-136.83	0.420
Pediatric versus adult	57.76	-81.48-197.02	0.386

CI, confidence interval; Coef, coefficient.

## References

[1] Matzinger P (1994). Tolerance, danger, and the extended family. Annu Rev Immunol.

[2] Roh JS, Sohn DH (2018). Damage-associated molecular patterns in inflammatory diseases. Immune Netw.

[3] Feldman P, Due MR, Ripsch MS (2012). The persistent release of HMGB1 contributes to tactile hyperalgesia in a rodent model of neuropathic pain. J Neuroinflammation.

[4] Abraham NG, Asija A, Drummond G (2007). Heme oxygenase-1 gene therapy: recent advances and therapeutic applications. Curr Gene Ther.

[5] Min HJ, Kim JH, Yoo JE (2017). ROS-dependent HMGB1 secretion upregulates IL-8 in upper airway epithelial cells under hypoxic condition. Mucosal Immunol.

[6] Min HJ, Ko EA, Wu J (2013). Chaperone-like activity of high-mobility group box 1 protein and its role in reducing the formation of polyglutamine aggregates. JImmunol.

[7] Schaefer L (2014). Complexity of danger: the diverse nature of damage-associated molecular patterns. J Biol Chem.

[8] Min HJ, Kim KS, Yoon JH (2017). T-helper 2 cytokine-induced heat shock protein 70 secretion and its potential association with allergic rhinitis. Int. Forum Allergy Rhinol.

[9] Min HJ, Kim SJ, Kim TH (2015). Level of secreted HMGB1 correlates with severity of inflammation in chronic rhinosinusitis. Laryngoscope.

[10] Min HJ, Yoon JH, Kim CH (2016). HSP70 is associated with the severity of inflammation in chronic rhinosinusitis. Am J Rhinol Allergy.

[11] Patel S (2018). Danger-associated molecular patterns (DAMPs): the derivatives and triggers of inflammation. Curr Allergy Asthma Rep.

[12] Qu B, Jia Y, Liu Y (2015). The detection and role of heat shock protein 70 in various nondisease conditions and disease conditions: a literature review. Cell Stress Chaperones.

[13] Shevchenko MA, Troyanova NI, Servuli EA (2016). Study of immunomodulatory effects of extracellular HSP70 in a mouse model of allergic airway inflammation. Biochemistry (Mosc).

[14] Ryu JH, Yoo JY, Kim MJ (2013). Distinct TLR-mediated pathways regulate house dust mite-induced allergic disease in the upper and lower airways. J Allergy Clin Immunol.

[15] Stroup DF, Berlin JA, Morton SC (2000). Meta-analysis of observational studies in epidemiology: a proposal for reporting. Meta-analysis Of Observational Studies in Epidemiology (MOOSE) group. JAMA.

[16] Liberati A, Altman DG, Tetzlaff J (2009). The PRISMA statement for reporting systematic reviews and meta-analyses of studies that evaluate healthcare interventions: explanation and elaboration. Ann Int Med.

[17] Hozo SP, Djulbegovic B, Hozo I (2005). Estimating the mean and variance from the median, range, and the size of a sample. BMC Med Res Methodol.

[18] Wan X, Wang W, Liu J, Tong T (2014). Estimating the sample mean and standard deviation from the sample size, median, range and/or interquartile range. BMC Med Res Methodol.

[19] Cavone L, Cuppari C, Manti S (2015). Increase in the level of proinflammatory cytokine HMGB1 in nasal fluids of patients with rhinitis and its sequestration by glycyrrhizin induces eosinophil cell death. Clin Exp Otorhinolaryngol.

[20] Di Candia L, Gomez E, Venereau E (2017). HMGB1 is upregulated in the airways in asthma and potentiates airway smooth muscle contraction via TLR4. J Allergy Clin Immunol.

[21] Seys SF, Hox V, Van Gerven L (2015). Damage-associated molecular pattern and innate cytokine release in the airways of competitive swimmers. Allergy.

[22] Shimizu S, Kouzaki H, Kato T (2016). HMGB1-TLR4 signaling contributes to the secretion of interleukin 6 and interleukin 8 by nasal epithelial cells. Am J Rhinol Allergy.

[23] Wood KL, Nunley DR, Moffatt-Bruce S (2010). The role of heat shock protein 27 in bronchiolitis obliterans syndrome after lung transplantation. J Heart Lung Transplant.

[24] Maile R, Jones S, Pan Y (2015). Association between early airway damage-associated molecular patterns and subsequent bacterial infection in patients with inhalational and burn injury. Am J Physiol Lung Cell Mol Physiol.

[25] van Zoelen MA, Ishizaka A, Wolthuls EK (2008). Pulmonary levels of high-mobility group box 1 during mechanical ventilation and ventilator-associated pneumonia. Shock.

[26] Jabaudon M, Blondonnet R, Roszyk L (2015). Soluble forms and ligands of the receptor for advanced glycation end-products in patients with acute respiratory distress syndrome: an observational prospective study. PLoS One.

[27] Ebina M, Taniguchi H, Miyasho T (2011). Gradual increase of high mobility group protein b1 in the lungs after the onset of acute exacerbation of idiopathic pulmonary fibrosis. Pulm Med.

[28] Ganter MT, Ware LB, Howard M (2006). Extracellular heat shock protein 72 is a marker of the stress protein response in acute lung injury. Am J Physiol Lung Cell Mol Physiol.

[29] Higa F, Furugen M, Koide M (2014). Clinical evaluation of high mobility group box 1 protein in *Legionella pneumophila* pneumonia. J Infect Chemother.

[30] Kanazawa H, Tochino Y, Asai K (2012). Validity of HMGB1 measurement in epithelial lining fluid in patients with COPD. Eur J Clin Invest.

[31] Lee EJ, Lim JY, Lee SY (2012). The expression of HSPs, anti-oxidants, and cytokines in plasma and bronchoalveolar lavage fluid of patients with acute respiratory distress syndrome. Clin Biochem.

[32] Muller MC, Tuinman PR, Vlaar AP (2014). Contribution of damage-associated molecular patterns to transfusion-related acute lung injury in cardiac surgery. Blood Transfus.

[33] Rong B, Cai X, Liu H (2016). Increased level of Hsp90-beta in bronchoalveolar lavage fluid correlates with lymphatic invasion and advanced stage of lung cancer patients. Am J Transl Res.

[34] Tasaka S, Kobayashi S, Kamata H (2010). Cytokine profiles of bronchoalveolar lavage fluid in patients with pneumocystis pneumonia. Microbiol Immunol.

[35] Zhang S, Zhao YF, Zhang MZ (2017). The diagnostic value of tumor markers in bronchoalveolar lavage fluid for the peripheral pulmonary carcinoma. Clin Respir J.

[36] Liou TG, Adler FR, Keogh RH (2012). Sputum biomarkers and the prediction of clinical outcomes in patients with cystic fibrosis. PLoS One.

[37] Salpietro C, Cuppari C, Grasso L (2013). Nasal high-mobility group box-1 protein in children with allergic rhinitis. Int Arch Allergy Immunol.

[38] Watanabe T, Asai K, Fujimoto H (2011). Increased levels of HMGB-1 and endogenous secretory RAGE in induced sputum from asthmatic patients. Respir Med.

[39] Hou C, Zhao H, Liu L (2011). High mobility group protein B1 (HMGB1) in asthma: comparison of patients with chronic obstructive pulmonary disease and healthy controls. Mol Med.

[40] Ferhani N, Letuve S, Kozhich A (2010). Expression of high-mobility group box 1 and of receptor for advanced glycation end products in chronic obstructive pulmonary disease. Am J. Respir Crit Care Med.

[41] Hamada N, Maeyama T, Kawaguchi T (2008). The role of high mobility group box1 in pulmonary fibrosis. Am J Respir Cell Mol Biol.

[42] Cuppari C, Manti S, Chirico V (2015). Sputum high mobility group box-1 in asthmatic children: a noninvasive sensitive biomarker reflecting disease status. Ann Allergy Asthma Immunol.

[43] Chirico V, Lacquaniti A, Leonardi S (2015). Acute pulmonary exacerbation and lung function decline in patients with cystic fibrosis: high-mobility group box 1 (HMGB1) between inflammation and infection. Clin Microbiol Infect.

[44] Sukkar MB, Wood LG, Tooze M (2012). Soluble RAGE is deficient in neutrophilic asthma and COPD. Eur Respir J.

[45] Xu H, Leng L, Chen M (2017). T cell subsets and cytokines are increased in the bronchoalveolar lavage fluid in children with pneumonia. Biomed Res.

[46] Jakubowska K, Naumnik W, Niklinska W (2015). Clinical significance of HMGB-1 and TGF-beta level in serum and BALF of advanced non-small cell lung cancer. Adv Exp Med Biol.

[47] Aksoy MO, Kim V, Cornwell WD (2017). Secretion of the endoplasmic reticulum stress protein, GRP78, into the BALF is increased in cigarette smokers. Respir Res.

[48] Hou C, Zhao H, Li W (2011). Increased heat shock protein 70 levels in induced sputum and plasma correlate with severity of asthma patients. Cell Stress Chaperones.

[49] Imbalzano E, Quartuccio S, Di Salvo E (2017). Association between HMGB1 and asthma: a literature review. Clin Mol Allergy.

[50] Corren J (2007). The connection between allergic rhinitis and bronchial asthma. Curr Opin Pulm Med.

[51] Bellussi LM, Cocca S, Passali GC (2017). HMGB1 in the pathogenesis of nasal inflammatory diseases and its inhibition as new therapeutic approach: a review from the literature. Int Arch Otorhinolaryngol.

[52] Cardoso AL, Fernandes A, Aguilar-Pimentel JA (2018). Towards frailty biomarkers: candidates from genes and pathways regulated in aging and age-related diseases. Ageing Res Rev.

[53] Enokido Y, Yoshitake A, Ito H (2008). Age-dependent change of HMGB1 and DNA double-strand break accumulation in mouse brain. Biochem Biophys Res Commun.

[54] Tang D, Kang RK, Xian W (2007). Nuclear heat shock protein 72 as a negative regulator of oxidative stress (hydrogen peroxide)-induced HMGB1 cytoplasmic translocation and release. JImmunol.

[55] Radons J (2016). The human HSP70 family of chaperones: where do we stand?. Cell Stress Chaperones.

[56] Taylor OJ, Thatcher MO, Carr ST (2017). High-mobility group box 1 disrupts metabolic function with cigarette smoke exposure in a ceramide-dependent manner. Int J Mol Sci.

