# Supplementary material for: Concentrations of HMGB1 and Hsp70 of healthy subjects in upper and lower airway: Literature Review and Meta-analysis
Source: Int J Med Sci. 2021 Feb 18;18(8):1760–7. doi: 10.7150/ijms.53500 (PMC7976589; doi:10.7150/ijms.53500)
Supplement: Supplementary file 1 — Supplementary materials. [file ijmsv18p1760s1.pdf]

Supplementary Information template:

SUPPLEMENTARY INFORMATION

TITLE: Concentrations of High-mobility Group Box 1 and Heat-shock Protein 70 in Upper and Lower Airways: Literature Review and Meta-analysis

Authors' full names: Hyun Jin Min, MD, PhD[1]; Kyung Soo Kim, MD, PhD[1]; Geun Joo Choi, MD, PhD[2]; Hyun Kang, MD, PhD[2]*; Fletcher A. White, MS, PhD[3]

Authors' affiliations:

[1] Department of Otorhinolaryngology-Head and Neck Surgery, Chung-Ang University College of Medicine, Seoul, Korea

[2] Department of Anesthesiology and Pain Medicine, Chung-Ang University College of Medicine, Seoul, Korea

[3] Department of Anesthesia, Indiana University School of Medicine, IN, USA

Appendix S1- Online-only Supplement

An ad-hoc table was designed to summarize the data from the included studies, show their key characteristics, and identify any important question associated with the aim of this meta-analysis. Continuous data were pooled, and the results were presented as mean values with a 95% confidence interval.

Heterogeneity among the studies was assessed using Cochrane's *Q* test, with significance defined by a $P < .10$ and I^2 statistics >50%. These thresholds indicated considerable heterogeneity according to the Mantel–Haenszel random-effects model. Otherwise, we applied the Mantel–Haenszel fixed-effects model.[1,2]

We conducted subgroup analyses based on the location from where the sample was drawn (upper airway vs. lower airway), sampling and analysis methods, the age category (paediatric vs. adult), and smoking history. Additionally, we performed meta-regression analysis to assess the influence of these variables on the normal reference ranges for the damage-associated molecular patterns (DAMPs). Univariate regression was performed if ≥10 studies reported the corresponding variable.

Sensitivity analyses were conducted to evaluate the influence of a single study on the overall effect estimated by excluding one study at a time when the heterogeneity was >50%. We performed sensitivity analyses in terms of sponsorship or conflict of interest.

If the number of combined studies showing substantial heterogeneity was <10, *t* statistics according to the Hartung–Knapp–Sidik–Jonkman method were used instead of the Z-test in all random-effects analyses in order to decrease the error rate.[3]

Medline

1. exp HMGB1 Protein/
2. ‘high mobility group b1 protein’.mp.
3. HMGB1.mp.
4. damage associated molecular pattern.mp.
5. DAMP.mp.
6. exp Heat-Shock Proteins/
7. exp HSP27 Heat-Shock Proteins/

8. HSP.mp.
9. exp HSP70 Heat-Shock Proteins/
10. exp HSP90 Heat-Shock Proteins/
11. or/1-10
12. airway.mp.
13. exp nose/
14. exp nasal mucosa/
15. nasal epithelium.mp.
16. nose epithelium.mp.
17. or/13-16
18. exp lung/
19. exp bronchoalveolar lavage/
20. exp bronchoalveolar lavage fluid/
21. BALF.mp.
22. or/18-21
23. 17 or 22
24. 11 and 23

EMBASE

1. ‘high mobility group b1 protein’/exp OR ‘high mobility group b1 protein’
2. Hmgb1
3. ‘damage associated molecular pattern’/exp
4. Damp

5. ‘heat shock protein’/exp
6. ‘heat shock cognate protein 70’/exp
7. ‘heat shock protein 27’/exp
8. ‘heat shock protein 90’/exp
9. hsp AND 27
10. hsp AND 70
11. hsp AND 90
12. #1 OR #2 OR #3 OR #4 OR #5 OR #6 OR #7 OR #8 OR #9 OR #10 OR #11
13. ‘lower respiratory tract’/exp
14. ‘lung’/exp
15. ‘lung lavage’/exp
16. BALF
17. bronchoalveolar AND lavage
18. #13 OR #14 OR #15 OR #16 OR #17
19. ‘upper respiratory tract’/exp
20. ‘nose’/exp
21. ‘nose epithelium’/exp
22. ‘nasal epithelial cell’/exp
23. #19 OR #20 OR #21 OR #22
24. #18 OR #23
25. #12 AND #24

Manuscript submission template:

References

[1]Jabaudon M, Blondonnet R, Roszyk L, Pereira B, Guérin R, Perbet S, Cayot S, Bouvier D, Blanchon L, Sapin V, Constantin JM. Soluble forms and ligands of the receptor for advanced glycation end-products in patients with acute respiratory distress syndrome: an observational prospective study. *PLoS One.* 2015; e0135857. https://doi.org/10.1371/journal.pone.0135857

[2]Ebina M, Taniguchi H, Miyasho T, Yamada S, Shibata N, Ohta H, Hisata S, Ohkouchi S, Tamada T, Nishimura H, Ishizaka A, Maruyama I, Okada Y, Takashi K, Nukiwa T. Gradual increase of high mobility group protein b1 in the lungs after the onset of acute exacerbation of idiopathic pulmonary fibrosis. *Pulm. Med.* 2011; **2011**: 916486.

[3]Ganter MT, Ware LB, Howard M, Roux J, Gartland B, Matthay MA, Fleshner M, Pittet JF. Extracellular heat shock protein 72 is a marker of the stress protein response in acute lung injury. *Am. J. Physiol. Lung Cell Mol. Physiol.* 2006; **291**: L354–61.
